# Repercussões da pandemia de COVID-19 nos serviços de saúde para pessoas com deficiência: relato dos profissionais de reabilitação

**DOI:** 10.1590/0102-311XPT223822

**Published:** 2023-06-26

**Authors:** Simone Vieira da Silva, Veronika Reichenberger, Gislene Inoue Vieira, Karina Aparecida Padilha Clemente, Vinícius Delgado Ramos, Christina May Moran de Brito

**Affiliations:** 1 Faculdade de Medicina, Universidade de São Paulo, São Paulo, Brasil.; 2 International Centre for Evidence in Disability, London School of Hygiene & Tropical Medicine, London, U.K.; 3 Prefeitura Municipal de São Paulo, São Paulo, Brasil.; 4 Hospital das Clínicas da Faculdade de Medicina da Universidade de São Paulo, São Paulo, Brasil.; 5 Instituto do Câncer do Estado de São Paulo Octavio Frias de Oliveira, São Paulo, Brasil.; 6 Hospital Sírio-Libanês, São Paulo, Brasil.

**Keywords:** Pandemia por COVID-19, Reabilitação, Atenção à Saúde, Pessoas com Deficiência, Serviços de Saúde para Pessoas com Deficiência, COVID-19 Pandemics, Rehabilitation, Delivery of Health Care, Disabled Persons, Health Services for Persons with Disabilities, Pandemia de la COVID-19, Rehabilitación, Atención a la Salud, Personas con Discapacidad, Servicios de Salud para Personas con Discapacidad

## Abstract

Diversos processos que permeiam a assistência à saúde, incluindo a reabilitação, precisam de brevidade para ser iniciados ou não podem ser interrompidos. Sendo assim, estes passaram por importantes adaptações durante a pandemia de COVID-19. Porém, não se sabe ao certo como os equipamentos de saúde adaptaram suas estratégias e quais foram os resultados. O estudo investigou como os atendimentos em reabilitação foram afetados durante a pandemia e quais foram as estratégias para a manutenção dos serviços prestados. Entre junho de 2020 e fevereiro de 2021, realizaram-se 17 entrevistas semiestruturadas com profissionais de saúde da área da reabilitação do Sistema Único de Saúde (SUS), que atuam em um dos três níveis de atenção, nas cidades de Santos e São Paulo, Estado de São Paulo, Brasil. Os discursos foram gravados, transcritos e analisados por meio da análise de conteúdo. Os profissionais relataram mudanças organizacionais em seus serviços, com a interrupção inicial dos atendimentos e, posteriormente, com a adoção de novos protocolos sanitários e o retorno gradativo dos atendimentos presenciais e/ou a distância. As condições de trabalho foram diretamente impactadas, pois houve necessidade de dimensionamento, capacitação, ampliação de carga horária, além da sobrecarga de trabalho e do esgotamento físico e mental dos profissionais. A pandemia determinou uma série de mudanças nos serviços de saúde, por vezes descontínuas, com a suspensão de inúmeros serviços e atendimentos. Alguns atendimentos presenciais foram mantidos, apenas para os pacientes que apresentavam risco de agravo em curto prazo. Medidas sanitárias preventivas e estratégias de continuidade dos atendimentos foram adotadas.

## Abstract (en)

Several healthcare processes, including rehabilitation, require prompt initiation and cannot be interrupted. Therefore, these processes underwent important adaptations during the COVID-19 pandemic. However, it is not fully known how healthcare facilities adapted their strategies and what the results were. This study investigated how rehabilitation services were affected during the pandemic and what strategies were employed to maintain the provided services. From June 2020 to February 2021, 17 semi-structured interviews were conducted with healthcare professionals working in rehabilitation services from the Brazilian Unified National Health System (SUS), who work at one of the three levels of care, in the municipalities of Santos and São Paulo, state of São Paulo, Brazil. The interviews were recorded, transcribed, and analyzed via content analysis. The professionals reported organizational changes in their services, with the initial interruption of appointments and, subsequently, the adoption of new sanitary protocols and the gradual return to in-person and/or remote appointments. Working conditions were directly impacted, as there was a need for staffing, training, increased workloads, as well as physical and mental exhaustion among professionals. The pandemic caused a series of changes in healthcare services, some of which were interrupted due to the suspension of numerous services and appointments. Some in-person appointments were maintained exclusively for patients who presented a risk of short-term deterioration. Preventive sanitary measures and strategies for continuity of care were adopted.

## Resumen

Diversos procesos que impregnan la asistencia a la salud, incluida la rehabilitación, deben iniciarse con prontitud o no pueden interrumpirse. Por lo que estos sufrieron importantes adaptaciones durante la pandemia de COVID-19. Sin embargo, no se sabe con certeza cómo las instalaciones de salud adaptaron sus estrategias y cuáles fueron los resultados. El estudio investigó cómo se vieron afectados los servicios de rehabilitación durante la pandemia y cuáles fueron las estrategias para mantener los servicios prestados. Entre junio del 2020 y febrero del 2021, se realizaron 17 entrevistas semiestructuradas con profesionales de la salud del área de rehabilitación del Sistema Único de Salud (SUS), que actúan en uno de los tres niveles de atención, en las ciudades de Santos y São Paulo, estado de São Paulo, Brasil. Los discursos se grabaron, se transcribieron y se analizaron mediante análisis de contenido. Los profesionales relataron cambios organizacionales en sus servicios, con la interrupción inicial de la atención y, posteriormente, con la adopción de nuevos protocolos sanitarios y el regreso gradual de la atención presencial o a distancia. Las condiciones de trabajo se vieron directamente afectadas, ya que fue necesario el dimensionamiento, la capacitación, la ampliación de la carga horaria, además de la sobrecarga de trabajo y del agotamiento físico y mental de los profesionales. La pandemia determinó una serie de cambios en los servicios de salud, en ocasiones discontinuos, con la suspensión de numerosos servicios y atenciones. Se mantuvieron algunas atenciones presenciales, solo para los pacientes con riesgo de agravamiento a corto plazo. Se adoptaron medidas sanitarias preventivas y estrategias de continuidad de las atenciones.

## Introdução

A pandemia de COVID-19 afetou diversos aspectos da vida humana e os sistemas de saúde em todo o mundo, além de causar uma crise econômica e social global. A velocidade e potencialidade na contaminação, aliadas à falta de compreensão inicial sobre o SARS-CoV-2, adicionaram mais complexidade e incertezas às estratégias de como lidar com a doença, intensificando os desafios à atenção em saúde e à proteção social das pessoas com deficiência (PcD) ^1^^,^^2^^,^^3^^,^^4^.

A assistência à saúde - especialmente alguns cuidados, como os de reabilitação, com processos que precisam ser iniciados imediatamente ou que não podem ser interrompidos - passou por importantes adaptações. O que antes era realizado de forma presencial precisou ser adaptado em razão do isolamento social. As tecnologias digitais de informação e comunicação (TDIC) se mostraram uma possibilidade de continuidade.

A abrangência e flexibilidade das TDIC ofereceram soluções inovadoras para a prestação de serviços de saúde com a realização de atendimentos remotos no contexto da pandemia. Os gestores, por sua vez, se depararam com desafios multifacetados, como o desencontro de informações, provocado pelas mudanças diárias de cenários, concomitante à necessidade de continuidade dos atendimentos, com atenção para a segurança de pacientes e dos profissionais ^5^^,^^6^^,^^7^^,^^8^^,^^9^^,^^10^.

Os termos telessaúde, teleconsultoria, teleorientação, teleconsulta, telediagnóstico, telemonitoramento, teleinterconsulta e teleducação ganharam novas discussões e diretrizes, exigindo rapidez na tomada de decisões, com necessidade de diversas adequações dos serviços de saúde em um cenário de desequilíbrio entre o avanço da doença, que se deu de forma exponencial, e a capacidade de resposta das organizações, que, por vezes, ocorreu em escala aritmética ^11^.

Cada espaço social se mostrou relativamente adepto às mudanças e foi afetado de diferentes maneiras. Desafios e descobertas foram compartilhados pelas equipes multidisciplinares, independentemente do nível de atenção à saúde a que pertenciam. Alternativas que pareciam temporárias e breves, para manutenção das intervenções, se converteram em novas modalidades de atuação diante das necessidades de saúde dos usuários, sendo implementadas para minimizar riscos adicionais à saúde, já muito fragilizada em alguns casos, sem prejudicar a continuidade dos tratamentos ^5^^,^^6^^,^^7^^,^^8^^,^^11^.

Algumas das adaptações sociais propostas durante a pandemia já eram parte das reivindicações das PcD sobre como a sociedade poderia melhor acolher as diversidades humanas. A possibilidade de trabalho remoto, adaptações nos locais de trabalho, oferta de serviços domiciliares, ampliação de políticas sociais, formas inclusivas de comunicação, qualificação das informações e prestação de serviços, no cotidiano das sociedades no pós-pandemia, promovem o exercício pleno e equitativo dos direitos humanos e liberdades fundamentais das PcD e de outras populações vulneráveis ^12^.

Nessa nova forma de conduzir os tratamentos e produzir cuidado, serviços de saúde por todo o mundo estão se reestruturando pelo risco de desorganização dos sistemas de saúde. Desde o início da pandemia, registraram-se quedas brutais nos números de consultas, exames e cirurgias e, consequentemente, aumento de mortes por outras enfermidades que não a COVID-19, como doenças cardiovasculares, câncer e outras doenças infecciosas. A falta de atendimento, somada ao receio da população de se expor à contaminação pelo vírus, além das recomendações dos órgãos de saúde de suspender os procedimentos eletivos, levou ao aumento da preocupação em relação aos diagnósticos primários e acompanhamentos ^13^^,^^14^^,^^15^.

A Organização Europeia para as Doenças Raras (EURORDIS) ^16^ anunciou resultados globais preliminares da primeira pesquisa multinacional sobre como a COVID-19 tem afetado pessoas que vivem com uma doença rara. Ela constatou que a pandemia dificultou o acesso aos cuidados: oito em cada dez pessoas tiveram suas consultas para terapias de reabilitação, como fonoterapia e fisioterapia (às vezes, as únicas acessíveis quando os demais tratamentos não estavam disponíveis), adiadas ou canceladas. De acordo com a Agência Nacional de Saúde Suplementar (ANS), no Brasil, houve redução de 33% na utilização dos serviços pelos beneficiários de planos de saúde nos quatro primeiros meses de 2020. As internações tiveram diminuição de quase 41% no mesmo período ^17^.

Nos sistemas públicos de saúde, essa tendência convoca a introdução de estratégias de gestão da clínica e de novos modelos de atenção à saúde baseados em evidências, especialmente os modelos inovadores de atenção às condições crônicas ^18^^,^^19^. No que tange aos cuidados às PcD, considerando os condicionantes sociais de saúde, com vistas à produção do cuidado integral, observa-se, no Estado de São Paulo, uma tentativa de substituição do paradigma do cuidado segmentado por especialidades por uma estratégia de cuidado em rede, com articulação dos diferentes níveis de atenção e prática interprofissional dialogada entre as especialidades ^20^.

A configuração do desenho regional da Rede de Cuidados à Pessoa com Deficiência (RCPcD) no estado, na lógica da Rede de Atenção à Saúde (RAS), permite que as redes organizadas contemplem os pontos de atenção previstos na *Portaria nº 793/2012* do Ministério da Saúde ^21^, embora com uma distribuição não equitativa. A organização não é baseada unicamente em torno dos centros especializados em reabilitação (CER), mas os engloba, buscando identificar diversos pontos da rede que realizam as ações de cuidados à PcD.

Com a relevância do cuidado de reabilitação e o contexto da pandemia, é importante investigar como os diferentes serviços de saúde adaptaram suas estratégias e os resultados que vêm colhendo de suas ações. Assim, este estudo apresenta uma análise sobre como os atendimentos realizados por profissionais que atuam na área da reabilitação, nos três níveis de atenção à saúde no Sistema Único de Saúde (SUS), foram afetados durante a pandemia e quais foram as estratégias utilizadas para a manutenção dos serviços prestados durante esse período.

## Método

Este estudo é parte da pesquisa *Fortalecendo a Inclusão de Pessoas com Deficiência no Sistema de Saúde no Brasil: Explorando o Acesso das Pessoas com Deficiência aos Serviços de Saúde nos Municípios de Santos e São Paulo*, que tem por objetivo avaliar o acesso das PcD ao SUS nessas localidades, a partir dos relatos das experiências de gestores, profissionais de saúde e PcD.

Para responder às questões deste estudo, optou-se pela pesquisa de abordagem qualitativa, com olhar para questões centradas na interpretação e na explicação da dinâmica das relações sociais ^22^. A interpretação dos dados se baseou na análise de conteúdo proposta por Bardin ^23^ e seguiu um processo rigoroso composto por pré-análise, exploração do material e tratamento dos resultados.

Neste estudo, foi utilizado um roteiro semiestruturado no qual havia uma sessão específica sobre a pandemia, com cinco questões: (a) como os serviços na sua unidade foram afetados pela pandemia?; quais atividades foram mantidas?; (b) como foram realizados os atendimentos a distância?; (c) alguns pacientes/usuários foram convocados para atendimentos presenciais?; (d) que estratégias foram adotadas como medidas de precaução na unidade?; e (e) foi dado apoio para outra unidade? Caso sim, de que forma?

As pesquisadoras entraram em contato com os responsáveis pelas unidades de saúde dos municípios de São Paulo e Santos (Estado de São Paulo), incluindo unidades básicas de saúde (UBS), núcleos de apoio à saúde da família (NASF), CER e hospitais. Seis deles demonstraram interesse em participar da pesquisa, sendo dois de cada nível de atenção. A gerência forneceu uma lista com o nome dos profissionais e seus contatos para que eles também fossem convidados a participar. Aqueles que se mostraram disponíveis foram entrevistados a distância, entre junho de 2020 e fevereiro de 2021, sendo suas falas gravadas, transcritas e analisadas.

A amostra foi composta por 17 participantes, dois médicos(as), dois terapeutas ocupacionais, três fonoaudiólogos(as), três psicólogos(as), dois enfermeiros(as), um(a) assistente social, quatro fisioterapeutas, dos três níveis de atenção, de três unidades de Santos e outras três de São Paulo (Quadro 1).

**Quadro 1:** Características gerais dos participantes. Santos e São Paulo, Estado de São Paulo, Brasil.

CIDADE	NÍVEL DE ATENÇÃO	INSTITUIÇÃO	PROFISSÃO	IDADE (EM ANOS)	SEXO	ANO DE FORMAÇÃO
Santos	Primário	NASF	Psicólogo(a)	38	Feminino	2008
			Assistente social	64	Feminino	1978
	Secundário	CER	Terapeuta ocupacional	30	Feminino	2012
			Fonoaudiólogo(a)	46	Feminino	1995
			Psicólogo(a)	31	Feminino	2011
			Fisioterapeuta	52	Masculino	2004
			Enfermeiro(a)	48	Feminino	2003
	Terciário	Hospital	Fisioterapeuta	37	Feminino	2014
			Fisioterapeuta	40	Feminino	2001
São Paulo	Primário	UBS	Psicólogo(a)	33	Masculino	2009
			Terapeuta ocupacional	51	Feminino	1996
	Secundário	CER	Enfermeiro(a)	36	Feminino	2003
			Fonoaudiólogo(a)	29	Feminino	2012
			Fonoaudiólogo(a)	53	Feminino	1989
			Fisioterapeuta	33	Feminino	2009
	Terciário	Hospital	Médico(a) - Geriatra	43	Masculino	2007
			Médico(a) - Ortopedista	65	Masculino	1980

CER: centro especializado em reabilitação; NASF: núcleos de apoio à saúde da família; UBS: unidade básica de saúde.

Fonte: elaboração própria (2022).

A partir da transcrição das entrevistas, foi realizado um *brainstorm* e criado um *codebook* com seis subcategorias, agrupadas em duas principais categorias, que nortearam toda a análise de dados (Quadro 2).

**Quadro 2:** Categorias do *codebook*.

CATEGORIAS	SUBCATEGORIAS	PROFISSIONAIS QUE ABORDARAM O TEMA
Serviços e atendimentos	Interrompidos	12 (8 em Santos; 4 em São Paulo)
	Mantidos	12 (8 em Santos; 4 em São Paulo)
Estratégias	Continuidade do atendimento	9 (6 em Santos; 3 em São Paulo)
	Medidas sanitárias preventivas	17 (9 em Santos; 8 em São Paulo)
	Telemonitoramento e teleconsulta	14 (7 em Santos; 7 em São Paulo)
	Articulação em rede	10 (4 em Santos; 6 em São Paulo)

Fonte: elaboração própria (2022).

Respeitando a *Resolução nº 466/2012* do Conselho Nacional de Saúde e suas complementares, este estudo foi submetido aos Comitês de Ética em Pesquisa do Hospital das Clínicas da Faculdade de Medicina da Universidade de São Paulo (CAAE: 26306519.4.0000.0065; parecer nº 4.038.259) e da Secretaria Municipal de Saúde de São Paulo (CAAE: 26306519.4.3001.0086; parecer nº 4.104.859). Os participantes assinaram o Termo de Consentimento Livre e Esclarecido, que apresentava os objetivos e detalhamentos da pesquisa, bem como assegurava a confidencialidade.

## Resultados e discussão

Em nossa amostra, a maioria dos profissionais que atuam na reabilitação são mulheres, com idade entre 29 e 65 anos, com ampla experiência na área e estão concentrados nos CERs. A análise foi desenvolvida a partir do *codebook*, em duas grandes categorias identificadas - serviços e atendimentos e estratégias -, resultando em seis subcategorias, que serão detalhadas a seguir.

### Medidas sanitárias preventivas

Com a pandemia de COVID-19, a rotina na saúde passou por mudanças significativas. Os profissionais relataram que as orientações partiram do Ministério da Saúde e das instituições. Embora a *Portaria nº 245/2020*^24^ tenha sido assinada em 24 de março de 2020, com orientações para suspender os atendimentos, alguns profissionais explicitaram a demora do poder público em estabelecer normas e orientações. Isso levou os profissionais a adotarem medidas preventivas segundo o que consideravam adequado, como relata uma fisioterapeuta: “*Eu já fui afastando aqueles pacientes que era possível de você dar alta. Eu fui dando alta. Aqueles pacientes que eram de grupos de risco e ofereciam menos, é* (...) *menos problemas de uma tonificação de lesão, eu optei por afastar*”. Sua fala expressa a forma como muitos profissionais atuaram na reabilitação nesse cenário: mensuravam o risco de perda funcional com a interrupção dos cuidados versus o risco de desenvolvimento de quadros mais graves de COVID-19, como no caso de pacientes frágeis, imunossuprimidos e obesos.

Com o aumento da demanda de pacientes sintomáticos nos primeiros meses, as unidades de saúde promoveram treinamentos para uso dos equipamentos de proteção individual (EPI), como touca, máscara descartável ou *face shield*, óculos de proteção, jaleco permanente, avental descartável, roupa privativa etc. Ocorreram alterações na forma dos atendimentos, com a criação de protocolos baseados no momento singular da pandemia. Considerando que parte significativa das pessoas que requerem reabilitação são mais vulneráveis, pacientes frágeis, idosos, imunossuprimidos e com diversas comorbidades ^25^^,^^26^^,^^27^, a maioria se sentia insegura para voltar aos atendimentos presenciais. Com isso, no início da pandemia, houve uma redução momentânea da demanda e foram implementadas medidas preventivas básicas, como intervalos mais longos entre as consultas, distanciamento físico, higienização mais frequente de mãos e equipamentos, além do uso de máscaras.

Os profissionais relatam que adotaram cuidados específicos para atender os pacientes, visando a menor exposição possível ao risco. Uma das alternativas foi deixá-los decidir se gostariam ou acreditavam ser necessário comparecer à unidade de saúde, sem impor o atendimento presencial. Quando possível, as decisões foram compartilhadas e tomadas em consenso, após o diálogo entre profissionais, pacientes e/ou familiares.

Houve redução do número de pacientes por horário e por sala, bem como dos profissionais atuantes. Para evitar aglomerações, as atividades em grupo foram canceladas. Caso os pacientes não tivessem máscaras, alguns serviços as forneciam. Avisos e cartazes com orientações foram espalhados pelas unidades. Uma terapeuta ocupacional de um CER relatou: “*Tivemos que reduzir muito o número de pacientes por horário e a quantidade de pacientes por sala (de um a dois só), dependendo do tamanho da sala*”. Uma enfermeira acrescentou: “*Naquele horário que você atendia três, passou a atender um. Então, querendo ou não, impactou. E o que acontece? O que fazia com três, agora faz com um de cada vez. Sem o* [atendimento] *grupal, você meio que breca a sua fila da demanda, então começa a acumular*”.

Os pacientes encaminhados para o CER pelo programa IntegraSUS ^28^ eram triados para avaliar a necessidade de comparecer presencialmente à unidade. Os profissionais relatam que no início da pandemia a prioridade era mantê-los em casa. Foram implementados cuidados com a estrutura e com o fluxo de pessoas circulando nas unidades, assim como controle da ocupação interna e do sistema de ventilação, para evitar exposição ao contágio. As unidades que atendiam simultaneamente os pacientes com e sem COVID-19 precisaram organizar um fluxo que promovesse uma separação segura entre eles.

Nos atendimentos às crianças, os profissionais relataram que convocavam os responsáveis, e a criança comparecia apenas nos casos mais graves. O atendimento interdisciplinar, composto por profissionais atuando simultaneamente, passou a ser realizado por uma equipe reduzida. Além disso, restringiu-se a quantidade de acompanhantes. Anteriormente à consulta presencial na unidade, eram realizadas ligações telefônicas, com o intuito de fazer uma triagem quanto à presença de sintomas de COVID-19 em pacientes e/ou familiares.

Nas unidades, profissionais aferiam a temperatura e orientavam quanto à higiene das mãos e ao uso de máscaras. Os atendimentos ficaram mais espaçados, individuais e com as medidas de higienização entre eles, tanto de material, ambiente, quanto dos EPIs, com descarte de parte deles. Para tanto, foram desenvolvidos alguns manuais de orientação e os profissionais de saúde adotaram as práticas recomendadas para evitar o contágio ^29^^,^^30^.

Serviços que continuaram a ser prestados, como vacinação e curativos, tinham número reduzido de pacientes e controle focado nas demandas. Os profissionais relataram ter utilizado muitas salas de isolamento, pois, em caso de suspeita, o paciente ficava isolado. Essa estratégia sobrecarregava a estrutura das unidades e foi considerada a maior mudança por ser incomum nos contextos avaliados. O distanciamento e o isolamento social e suas consequências são pouco estudados por ser medidas emergenciais, o que torna seus efeitos desconhecidos ^31^.

Antes das consultas, os profissionais verificavam a possibilidade de o paciente comparecer sozinho ou com apenas um acompanhante para assegurar o distanciamento na recepção. Nas salas de espera, havia orientação para os pacientes se sentarem em cadeiras intercaladas, com o objetivo de evitar aproximação e possíveis contágios, seguindo as orientações estaduais sobre o distanciamento social, conforme o *Decreto nº 64.881/2020*^32^.

Segundo os entrevistados, na terapia ocupacional e na fisioterapia o distanciamento social foi mais difícil, pois muitas vezes eles precisavam orientar o posicionamento ou manipular o paciente. Esse ponto também foi relatado pelos médicos, tendo em vista a necessidade do exame físico.

Os pacientes que precisavam receber seus aparelhos auditivos receberam ligações que verificavam a possibilidade de seu comparecimento ao CER e explicavam que a unidade estava com atendimentos reduzidos e agendados de acordo com a disponibilidade e o interesse do paciente. A maioria preferiu receber presencialmente os aparelhos e foi orientada a ir, preferencialmente, com veículo próprio ou não coletivo, com apenas um acompanhante, principalmente porque o público majoritário era de idosos. Embora essa orientação expresse a preocupação com o risco imposto pela exposição ao vírus, ela desconsidera que muitas pessoas, incluindo boa parte das PcD, que já estavam em vulnerabilidade social sofreram profundos agravos durante a pandemia ^33^.

Houve, ainda, situações polarizadas nos serviços de saúde. Um hospital em Santos funcionou como hospital de campanha, com uma unidade de terapia intensiva (UTI) para pacientes com COVID-19. Um dos profissionais relatou que um CER em São Paulo foi dedicado exclusivamente aos pacientes de reabilitação e era chamado de unidade limpa, pois não atendia de forma alguma pacientes com sintomas de COVID-19. As medidas sanitárias preventivas estiveram entre os assuntos prioritários durante as entrevistas, sendo mencionadas por todos os participantes.

### Serviços e atendimentos interrompidos

A primeira medida implementada foi cancelar a maior parte dos atendimentos, principalmente da população considerada vulnerável (crianças, gestantes e idosos; e pacientes ou profissionais, com comorbidades) ^25^^,^^26^^,^^27^. Segundo os profissionais, foram cancelados os atendimentos presenciais, como os grupos de crianças, reabilitação respiratória, reabilitação geral, reabilitação intelectual e neurológica, grupos educativos, de monitoramento glicêmico, de tabagismo, de orientação nutricional e de gestantes. Uma psicóloga do CER confirmou: “*tanto para as crianças quanto para os adultos a gente tem evitado. Os atendimentos que aconteciam às vezes de um paciente junto com outro, em dupla, ou às vezes em grupos, todos esses tipos de atendimentos foram todos suspensos*”.

Os atendimentos da atenção básica foram suspensos e hipertensos e diabéticos já não tinham mais suas consultas garantidas. As visitas domiciliares também foram suspensas, afetando a vigilância dos agentes comunitários de saúde (ACS) por alguns meses. Ainda assim, o fornecimento das medicações de uso contínuo foi mantido. Nos hospitais, foram interrompidos os atendimentos a pacientes com indicação de cirurgias eletivas.

No início da pandemia, os serviços de plantão no CER para manutenção de aparelhos auditivos foram suspensos e os exames foram desmarcados e voltaram a ser realizados após um tempo e com adaptações (agendamento e restrição na quantidade de pacientes) para manter o cuidado e controle das pessoas no acesso às unidades de reabilitação, seguindo as normas estabelecidas do distanciamento social para populações vulneráveis, incluindo as PcD ^34^.

### Serviços e atendimentos mantidos

Durante a pandemia, os profissionais relataram que as unidades mantiveram alguns atendimentos sem interrupção. Com várias adaptações, os atendimentos presenciais foram mantidos para os pacientes que apresentavam risco de agravo em curto período, como pós-operatórios ortopédicos; com afecções neurológicas agudas, como quadros recentes de acidente vascular cerebral pós-alta hospitalar; pós-cirúrgicos, como sequelas de traumas; assim como quadros osteomioarticulares agudos com demandas de reabilitação. Os casos que apresentavam necessidades mais urgentes não deixaram de ser atendidos, visando minimizar as sequelas, como explicou uma fisioterapeuta do CER: “*mantivemos uma atividade, assim, mais da basal, daqueles que realmente, assim, aconteceria algum, enfim, algum prejuízo na recuperação dele*”.

Foram mantidos atendimentos a recém-nascidos de risco, crianças com até dois anos e pré-natal, vacinação, curativos, consultas de emergência, com quantidade diária limitada, porém o foco dos atendimentos era o cuidado a pacientes com sintomas de COVID-19. Uma fisioterapeuta de um hospital afirmou: “*mantivemos o atendimento do hospital normalmente* (...) *como parar de atender, principalmente maternidade? A gente manteve o serviço de saúde*”. Uma fisioterapeuta do CER acrescentou: “*a gente monitorou com um pouco mais afinco os bebês de risco, a gente tem grupos grandes de bebês de risco em relação ao desenvolvimento, porque pra eles um, dois meses faria muita diferença*”.

### Estratégias de continuidade do atendimento

Para a continuidade dos serviços e atendimentos que precisaram ser mantidos por sua importância e para a retomada daqueles que precisaram ser cancelados, as unidades implementaram estratégias que foram estabelecidas por normas governamentais ou definidas de acordo com as especificidades de cada local. Entre as estratégias citadas pelos profissionais estão várias mudanças na forma de atendimento. As equipes formaram turmas de intervenção, passando a trabalhar em plantões e a fazer monitoramento por telefone, e-mail ou alguma forma de contato para que os profissionais pudessem fornecer orientações e estimular a prática de atividades, como no caso de famílias com criança com autismo ou deficiência intelectual, colocando-se à disposição para tirar dúvidas. Como relatou uma fisioterapeuta: “*a gente mandou para as mães umas cartilhas de desenvolvimento motor pra tentar estimular em casa com algumas atividades, a gente foi enviando essas cartilhas e algumas instruções pra elas tentarem fazer em casa e tirando as dúvidas sempre por telefone*”.

Quando houve uma flexibilização, os profissionais convocaram os pacientes que apresentavam urgência e contataram os pais para avaliar a evolução das crianças durante o período de monitoramento a distância para elaborar intervenções para os casos com maior demanda de cuidado. Os atendimentos não eram semanais como antes, e sim espaçados, com um intervalo maior de tempo, permitindo que as famílias expressassem sentimentos de segurança para comparecer ou não às unidades. Os casos que não apresentavam urgência permaneceram no telemonitoramento.

No retorno gradual dos atendimentos, estes começaram a ser realizados individualmente, sem previsão para retorno dos grupos. No caso das visitas domiciliares, a equipe não deixou de atuar, porém não entrava nas casas. Nas unidades que permitiam o acesso sem encaminhamento e ofereciam plantão para manutenção de aparelhos auditivos, os serviços passaram a ser agendados e planejados para evitar aglomeração. Uma profissional que atua no CER de Santos referiu que, antes da pandemia, não havia fila de espera por atendimento e, durante a pandemia, a fila voltou a crescer.

### Telemonitoramento e teleconsulta

No início da pandemia, o telemonitoramento enfrentava dificuldades de disponibilidade de recursos e do registro do faturamento do atendimento pelo Ministério da Saúde. Embora não houvesse um protocolo estruturado com orientações e equipamentos suficientes para a prática do teleatendimento, os profissionais passaram a contatar cada família e verificar como cada uma conseguia se adaptar. Uma psicóloga relatou: “*os atendimentos, eles foram passados pra teleatendimento, pra monitoramento via telefone. Depois de um tempo, né, do início da pandemia chegaram algumas webcams, então, alguns profissionais da unidade começaram a fazer atendimentos por vídeo*”.

O teleatendimento foi o principal meio de comunicação para pacientes da reabilitação infantil. Os profissionais relatam que passavam orientações e atividades *online*, por e-mail e/ou por telefone. Em algumas unidades, desde o primeiro mês da pandemia, foram realizados monitoramentos via telefone, semanalmente ou mensalmente, de todos os pacientes e, se houvesse necessidade, havia convocação para um atendimento presencial. As famílias se sentiram acolhidas, em especial os pais de crianças em reabilitação, por permanecerem em contato com os profissionais.

Quando os atendimentos foram liberados para o retorno presencial, o(a) assistente social ligava para os pacientes para verificar os motivos de sua ausência nas consultas. Durante o período da pandemia, os pacientes não foram desligados por falta, os profissionais buscavam compreender a situação de cada um antes de tomar qualquer decisão. Entre os desafios mais comuns citados, na tentativa de contato com os pacientes, estava a limitação dos pacientes em relação às TDIC e a desatualização dos números de telefone. Sobre as dificuldades enfrentadas no teleatendimento, um médico compartilhou sua experiência: “*o atendimento a distância foi feito, mais limitado e era difícil, não era uma consulta agendada, não foi uma coisa tão bem estruturada assim. A gente enfrenta alguns tipos de desafios na telemedicina por conta das pessoas que estão do outro lado da linha, que elas não conhecem o serviço, não estão estruturadas a utilizar*”.

O telemonitoramento foi eficiente para alguns grupos específicos, como os acamados e os de menor complexidade - hipertensos, diabéticos e os inscritos no Bolsa Família -, pois os profissionais conseguiam monitorar o acesso à medicação e o estado de saúde do paciente e dos familiares/cuidadores. Foram criados grupos no aplicativo WhatsApp para transmitir orientações e vídeos educativos sobre a pandemia e sobre medidas educativas para demandas frequentes de cuidado.

### Articulação em rede

Em Santos, uma das unidades funcionou como hospital de campanha, como já mencionado. Profissionais da enfermagem foram deslocados para fazer testes de COVID-19 em unidades abertas e para atender os casos de contaminação. Algumas unidades receberam doações de máscaras e álcool em gel, o que contribuiu para a redução da contaminação e segurança dos pacientes. O CER de Santos passava por reforma quando a pandemia se instalou, resultando na transferência de profissionais e pacientes para outras unidades.

Uma UBS em São Paulo instaurou uma equipe móvel multidisciplinar para apoiar os profissionais da linha de frente. Um dia em cada unidade, eram promovidos exercícios de alongamento, técnicas de relaxamento, momentos de conversas. Além disso, a equipe ajudou na confecção de máscaras para doação e na organização de filas. Sobre sua experiência durante o período da pandemia, um psicólogo relatou: “*em relação ao trabalho de psicólogo, a gente teve uma mudança também nesses meses de que foi feito o suporte ao colaborador. Suporte emocional. Então eu fui deslocado também para uma outra unidade, além da que eu já atendo, pra poder fazer o atendimento de acolhimento e um trabalho terapêutico com os colaboradores*”.

Alguns aspectos foram abordados em mais de uma das subcategorias, como “*atendimentos cancelados*”, que foi destaque em “*medidas sanitárias preventivas*” e “*serviços e atendimentos interrompidos*”. O controle do fluxo nas unidades apareceu como “*medidas sanitárias preventivas*” e “*estratégias de continuidade do atendimento*”.

As medidas sanitárias preventivas e as estratégias de continuidade do atendimento foram essenciais para o cuidado com os pacientes para que houvesse menor exposição ao contágio e redução nos prejuízos à saúde da população. O SUS esteve no centro da pandemia de COVID-19, com seus profissionais atuando nas frentes de prevenção, diagnóstico, tratamento e reabilitação ^1^.

Não foi exclusividade do Brasil cancelar serviços de saúde e manter apenas os atendimentos essenciais. Países da Europa suspenderam internações para reabilitação, autorizaram alta precoce e diminuíram as atividades assistenciais. Na Itália, Bélgica e Reino Unido, o cancelamento das atividades ambulatoriais atingiu 87%, resultando em milhões de pacientes, incluindo PcD, prejudicados. Mais gravemente, as PcD corriam risco de ser dispensadas dos cuidados prioritários em meio a uma pandemia, em uma situação contingencial, explicitando a inequidade de acesso ^35^.

A falta de coordenação de cuidados dos serviços territorializados e o diálogo limitado entre os serviços especializados e os de atenção básica no período da pandemia evidenciaram um vazio assistencial no que tange aos cuidados crônicos das PcD, uma população negligenciada, mesmo antes da pandemia ^1^. Medidas severas de proteção e prevenção, como distanciamento social e racionamento de saúde, baseadas em critérios discriminatórios, são estratégias que podem colaborar para a exclusão e para o aumento da vulnerabilidade, perpetuando barreiras atitudinais, ambientais e institucionais ^12^.

O teleatendimento se destacou por ser eficaz e seguro para a comunicação entre profissionais e pacientes, podendo ainda ser combinado com a modalidade presencial. A pandemia impôs o desafio da implementação da telemedicina no atendimento aos pacientes que, no caso das PcD, vivenciam a invisibilização, o confinamento ao longo do tempo e uma rotina de necessidades de cuidado em saúde ^1^^,^^12^.

Durante a pandemia, a saúde física e mental dos profissionais de saúde foi prejudicada devido a sobrecarga de trabalho, riscos de infecção, redistribuição constante e inesperada para novas funções, restrições generalizadas, avaliações frequentes de desempenho e preocupações com seu bem-estar e de seus familiares. Com isso, episódios de esgotamento, ansiedade e depressão entre os profissionais de saúde nesse período aumentaram significativamente ^36^^,^^37^. À medida que os casos de COVID-19 aumentavam, os recursos humanos e materiais se tornavam mais escassos ^1^.

Os profissionais relatam que não existiu interrupção nos serviços de saúde (apenas adequação e diversificação das atividades) e, como o número de contaminados aumentava a cada dia, houve a intensificação do trabalho e a necessidade de adaptações de forma dinâmica. Sobre o trabalho intenso, uma fonoaudióloga relatou: “*nós, funcionários da saúde, fomos impedidos inclusive de tirar férias, licenças, enfim, a saúde continuou trabalhando*”. Os profissionais lidaram com uma situação desconhecida e aprenderam com a prática. À medida que protocolos e estratégias eram elaborados, eles atuavam de forma intensa e contínua. Os profissionais descreveram o período como emocionalmente desgastante, relatando medo e angústia, e destacaram a importância do apoio da equipe. Uma fonoaudióloga compartilhou: “*no começo foi um pouco assustador, deu uma desestabilizada*”. Alguns profissionais faziam parte de grupos vulneráveis e passaram a trabalhar de forma remota, já outros precisaram ser afastados ao apresentarem sintomas de COVID-19. O medo da infecção, a escassez de EPIs e o excesso de trabalho impactaram de forma negativa a saúde física e mental dos profissionais de saúde, especialmente daqueles que atuaram na linha de frente no combate à pandemia de COVID-19 ^37^.

A Organização Mundial da Saúde (OMS) apresentou recomendações específicas de prevenção, controle, assistência e proteção social para as PcD que tiveram sua vulnerabilidade ampliada durante a pandemia. Mesmo em países que adotaram a *Convenção sobre os Direitos das Pessoas com Deficiência*^38^, como no Brasil, houve dificuldades para abordar as necessidades desse público adequadamente ^12^.

## Conclusões

Nossos resultados contribuem para a compreensão de como ocorreram os atendimentos de reabilitação para PcD em todos os níveis de cuidado durante a pandemia de COVID-19. Uma das primeiras ações foi suspender a maior parte dos atendimentos, principalmente para a população considerada mais vulnerável. Com várias adaptações, alguns atendimentos presenciais foram mantidos, apenas para os pacientes que apresentavam risco de agravo em curto prazo, visando minimizar as perdas funcionais e sequelas. Algumas das medidas sanitárias preventivas incluíram uso de EPIs, distanciamento social, triagem de sinais e sintomas e o intervalo mais longo entre os atendimentos. Para não provocar aglomeração, as atividades grupais foram canceladas. Outras ações foram o aumento de monitoramento por telefone e o uso das TDIC. Para os profissionais de saúde, o período da pandemia foi física e emocionalmente desgastante, com relatos de sentimentos de medo e angústia. Com relação à articulação da rede, ocorreu o deslocamento de profissionais para outras unidades, apoiando a realização de testes de COVID-19 e o cuidado às pessoas afetadas pelo vírus.

### Limitações

Existem pontos fortes e limitações neste estudo, que devem ser considerados na interpretação dos achados. A amostra por conveniência não reflete, em sua totalidade, a classe dos profissionais de reabilitação. O questionário não era específico sobre a COVID-19, mas sim uma sessão de um questionário mais amplo, o que dificultou o aprofundamento em alguns assuntos. As entrevistas foram realizadas durante um período em que as equipes estavam sobrecarregadas devido à pandemia. Por isso algumas entrevistas foram mais curtas do que o esperado, em razão da falta de disponibilidade. Embora medidas tenham sido implementadas na tentativa de reduzir o viés, como sugerir durante o agendamento que as entrevistas fossem feitas fora do ambiente de trabalho, algumas ocorreram durante os intervalos dos profissionais, enquanto estavam em seus locais de trabalho.

Para estudos futuros, sugerimos que a singularidade da vivência da deficiência durante o período de COVID-19 seja investigada com mais profundidade, juntamente às PcD, bem como as repercussões na assistência à saúde, com o objetivo de fomentar estratégias necessárias de atenção à saúde, potencializando, assim, o cuidado continuado e integrado.
